# Supplementary material for: A Framework for Annotation of Antigen Specificities in High-Throughput T-Cell Repertoire Sequencing Studies
Source: Front Immunol. 2019 Sep 26;10:2159. doi: 10.3389/fimmu.2019.02159 (PMC6775185; doi:10.3389/fimmu.2019.02159)
Supplement: Supplementary file 1 [file Data_Sheet_1.pdf]

Supplementary Figures and Tables for “A framework for annotation of antigen specificities in high-throughput T-cell repertoire sequencing studies”

Mikhail V Pogorelyy Mikhail Shugay

Supplementary Figure 1. VDJdb annotation results for B35+ and CMV+ samples compared to pooled control dataset. Box plots of the number of reads associated with annotated TCRs for various epitope specificities. Epitope specificities are encoded as the restricting HLA (e.g. B*35) and first three amino acids of the epitope sequence (e.g. IPS). Boxplots are colored according to the parent species of the epitope. Grey boxplots show the frequency in pooled control samples. VDJdb annotation is performed with 1 amino acid substitution allowed for CDR3aa.

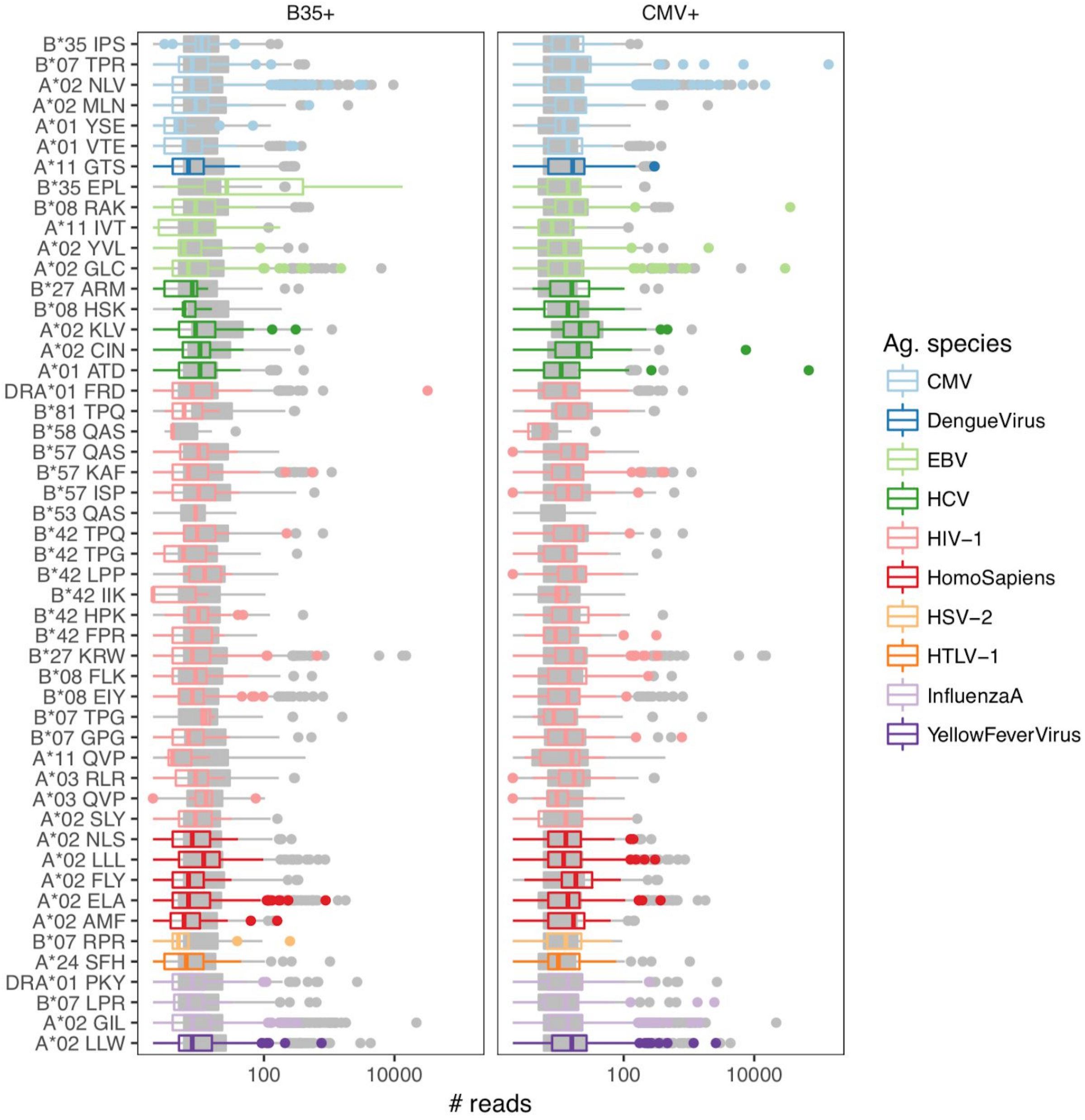

Supplementary Figure 2. Epitope-specific TCR frequencies in B35+, CMV+ and pooled control samples for selected epitopes. Boxplots show TCR frequency in the sample. Epitopes were filtered according to donor status and HLA restriction: HLA-B*35 for B35+ sample and CMV+/HLA-B*07 or HLA-A*02 for CMV+ sample. Boxplots are colored by parent species of the epitope, grey boxplots show the TCR frequency in pooled control samples.

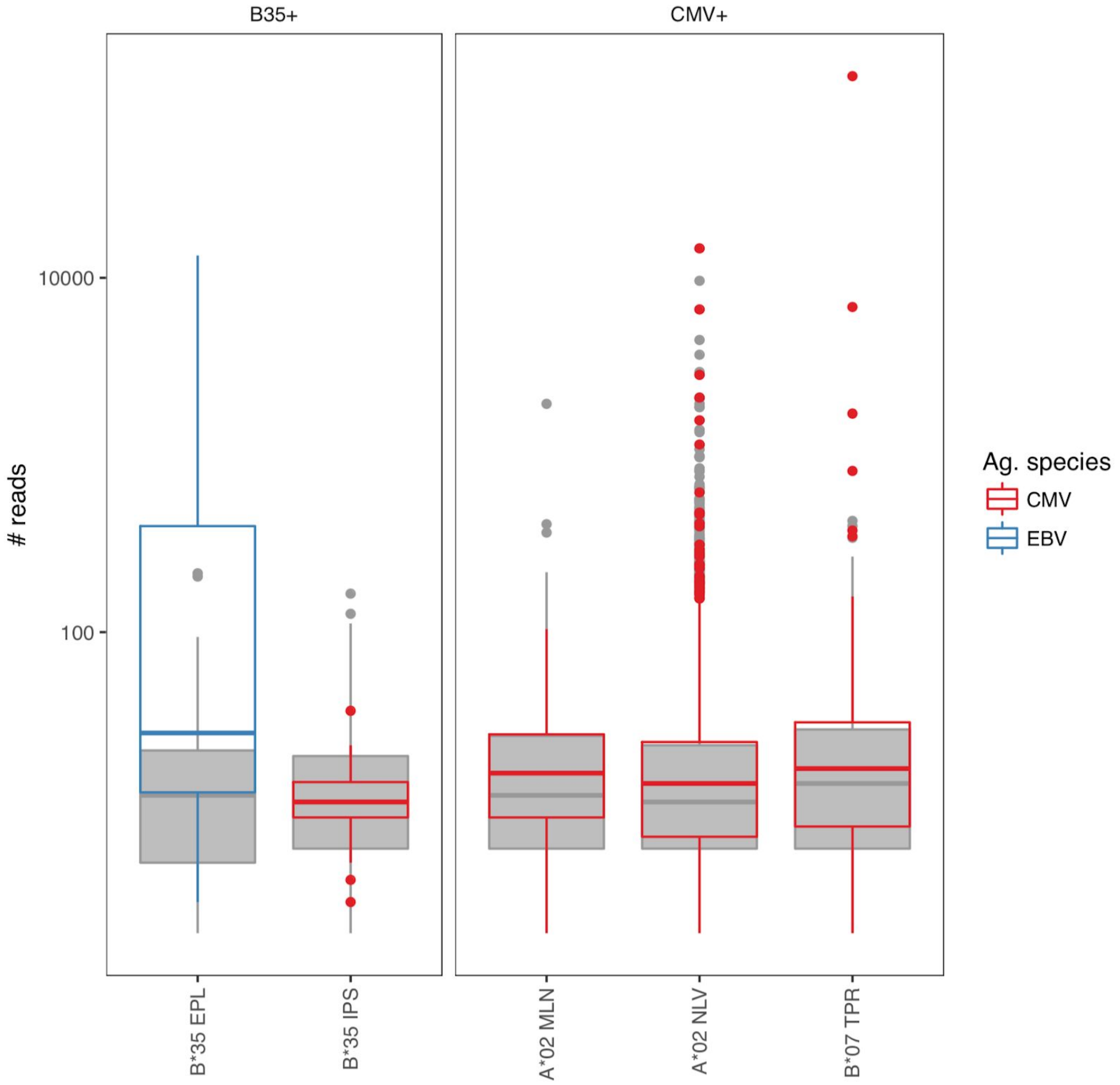

Supplementary Figure 3. Comparing enrichment fold to TCR expansion rate. Scatter-plot comparing fraction of observed and expected neighbor sequences (enrichment fold, computed using TCRNET algorithm) and the T-cell expansion (TCR frequency in sample). Each point represents a TCR clonotype, points are covered by the enrichment fold P-value (TCRNET test). Significant correlation is observed for both B35+ (Spearman R=0.42, P=3x10^{-6}) and CMV+ (Spearman R=0.31, P=8x10^{-3}) samples.

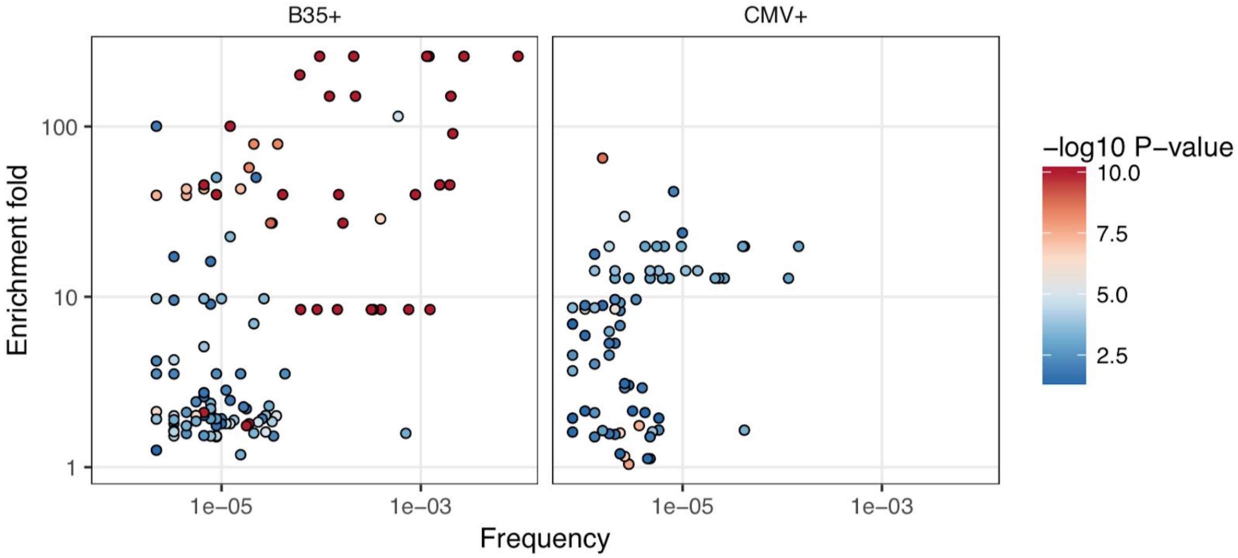

Supplementary Figure 4. Expression of specific TCRs in CMV+ samples. Mean TCR clonotype size (number of reads) for 28 A*02+B*07+CMV+ samples from Emerson et al dataset. Mean specific clonotype size is shown with red dots, red dashed line shown mean value across all samples. Black dashed line shown CMV+ clonotype size in the pool of 4 A*02+B*07+CMV- control samples used in the study (see main text). A*02:NLV and B*07:TPR epitopes are considered. A significant increase in specific T-cell clonotype size is observed: T statistic of 8.4 ($P=7x10^{-9}$) and T=7.0 ($P=2x10^{-7}$) respectively, single sample T-test comparing to control mean.

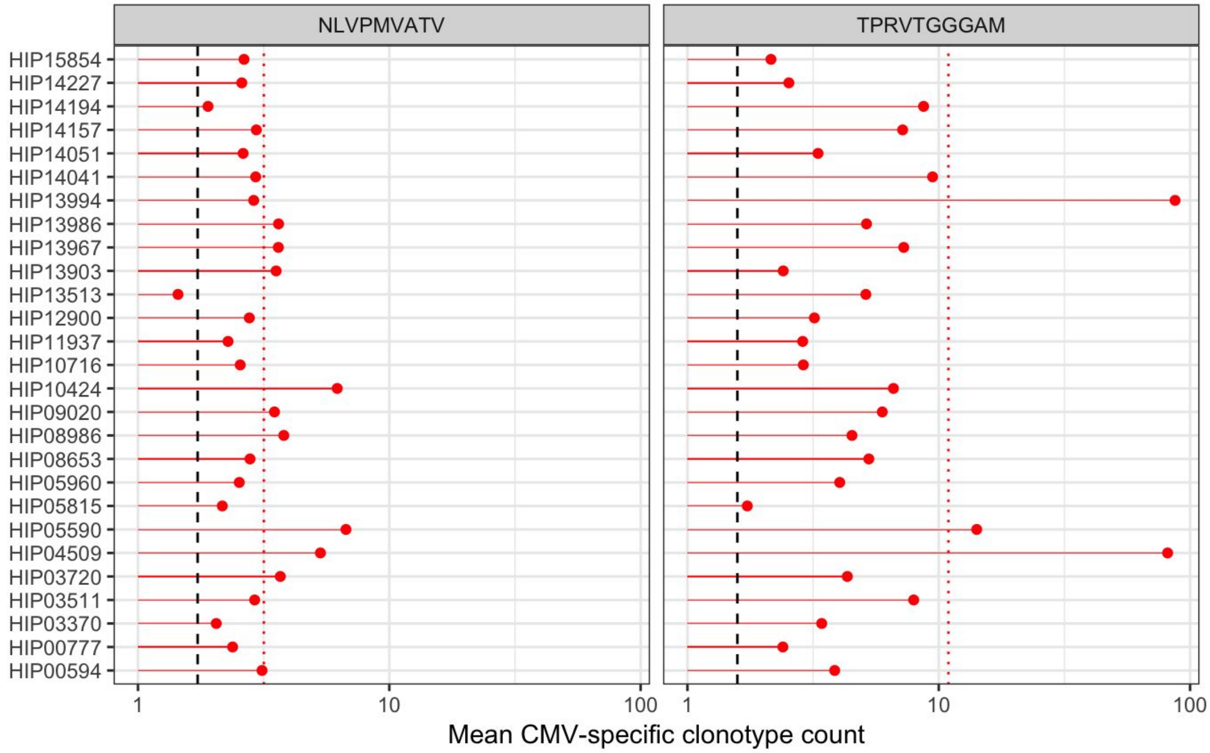

Supplementary Figure 5. Specific TCR enrichment in TCRNET core clonotypes. Fraction of unique TCR sequences annotated for CMV specificity (either A*02:NLV or B*07:TRP epitope) for original (full) and the set of TCRNET cores (tcrnet). TCRNET cores are specified as TCRs enriched in neighbours according to the TCRNET test with an adjusted P-value threshold of 0.05 (same as other analysis in the main text). A significant enrichment in the fraction of CMV-specific TCRs is observed for TCRNET dataset: T-statistic of 9.9 ($P=2x10^{-10}$) and T=5.4 ($P=10^{-5}$) for A*02:NLV and B*07:TRP respectively, paired T-test.

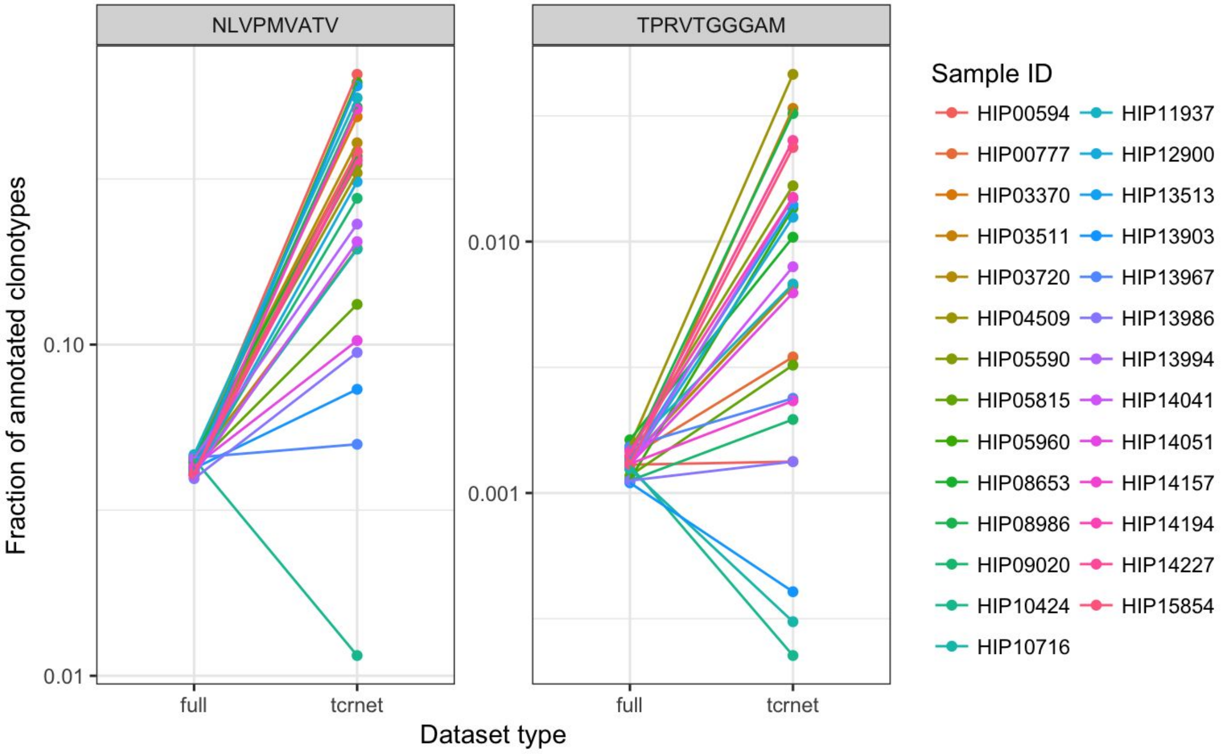

Supplementary Table 1. TCRNET clusters across top 10 most frequent specific TCRs annotated according to VDJdb. CMV-specific clonotypes with A*02 and B*07 HLA restriction are shown with corresponding number of reads. In case a TCR clonotype belongs to an inferred TCR cluster a cluster ID is provided.

HLA	CDR3aa	Reads	Frequency	Cluster ID
A*02	CASSLGQDTQYF	14664	0.384%	
	CASSSVNEQFF	6633	0.174%	
	CASLQGNTEAFF	2834	0.074%	
	CASSSVGGYTF	2110	0.055%	
	CASSLAGYEQYF	1570	0.041%	1
	CASSPTGNYGYTF	1146	0.030%	
	CASSQEGSQPQHF	615	0.016%	
	CASSYSADTGELFF	473	0.012%	
	CASSLDILSYNEQFF	472	0.012%	
	CASSLAPGATNEKLFF	463	0.012%	
B*07	CASSLQTGLNTEAFF	137472	3.599%	
	CASSPSRNTEAFF	6846	0.179%	
	CASSPHRNTEAFF	1713	0.045%	
	CASSFRQGIDTGELFF	813	0.021%	
	CASSYSSGELFF	375	0.010%	
	CASSYSHGELFF	348	0.009%	
	CASSYSRNTEAFF	286	0.007%	11
	CASSLRDGINTGELFF	159	0.004%	
	CASSLRQGANTGELFF	154	0.004%	
	CASSYSRLNTEAFF	133	0.003%	
